# Kinetics and Thermodynamics Studies for Cadmium (II) Adsorption onto Functionalized Chitosan with Hexa-Decyl-Trimethyl-Ammonium Chloride

**DOI:** 10.3390/ma13235552

**Published:** 2020-12-05

**Authors:** Cristina Ardean, Mihaela Ciopec, Corneliu Mircea Davidescu, Petru Negrea, Raluca Voda

**Affiliations:** Faculty of Industrial Chemistry and Environmental Engineering, Politehnica University of Timisoara, 2 Piata Victoriei, RO 300006 Timisoara, Romania; cristina.ardean@student.upt.ro (C.A.); mihaela.ciopec@upt.ro (M.C.); raluca.voda@upt.ro (R.V.)

**Keywords:** chitosan functionalization, cadmium adsorption, hexa-decyl-trimethyl-ammonium extractant, adsorption capacity

## Abstract

A new adsorbent material was obtained by functionalization of chitosan with hexa-decyl-trimethyl-ammonium chloride and tested as an adsorbent for Cd(II) ions. Functionalization is due to the desire to improve the adsorbent properties of the biopolymer used for removal of metallic ions. Obtained material was characterized by FTIR (Fourier Transform InfraRed spectroscopy), SEM (Scanning Electron Microscopy) and EDX (Energy dispersive X-ray Spectroscopy). To prove the Cd(II) adsorption mechanism, we performed adsorption tests determining influence of biopolymer ratio, pH, contact time, temperature and Cd(II) initial concentration. Obtained experimental data were modeled using two kinetics models: pseudo-first-order and pseudo-second-order models. Cd(II) adsorption kinetics was better described by pseudo-second-order model. Further, experimental data were fitted using three different adsorption isotherms: Langmuir, Freundlich and Sips. The studied adsorption process is well described by the Sips adsorption isotherm, when the maximum adsorption capacity value is near the experimental one. Likewise, we evaluated the values of thermodynamic parameters which indicate that the studied process is an endothermic and spontaneous one, being a physical adsorption. Prepared adsorbent materials have a maximum adsorption capacity of 204.3 mg Cd^2+^ per gram at pH > 4.0 and 298 K. In addition, this material was reused for Cd^2+^ recovery for 20 times.

## 1. Introduction

Cadmium is a heavy metal (soft and ductile, silvery white with bluish color, lustrous and electropositive properties [1]) which is primarily used in batteries, electroplating systems, stabilizers and pigments, fertilizers, as a neutron absorber in nuclear power plants and in the form of different salts as important constituents of many alloys [2,3]. In this context, from an environmental and occupational point of view, cadmium is considered a highly toxic component. Experimental data confirmed that once ingested Cd has a long half-life because of its low excretion rate. In this condition, prolonged exposure to Cd has toxic effects due to its accumulation in kidneys, liver, central nervous system and peripheral neuronal systems [4].

Word Health Organization’s International Agency for Research on Cancer and the United States National Toxicology Program designated cadmium as being a carcinogen for humans [2,5,6]. Different studies reveal that the exposure to Cd and Cd compounds is linked with the occurrence of pulmonary cancer [2,5,6]. Different studies performed have indicated that cadmium plays an important role in the human prostatic and renal cancers, while a small number of studies linked cadmium exposure with occurrence of liver, hematopoietic system, urinary bladder and stomach cancers [2,5,6]. Epidemiological and experimental studies demonstrate that Cd exposure affects the function of the nervous system, being associated with the presence of different symptoms (vertigo, headache, olfactory dysfunction, parkinsonian-like symptoms) [2,4,5,6,7].

Performed studies have revealed that Cd clearly represents a multi-tissue animal carcinogen. In rats, studies have revealed that the presence of pulmonary adenocarcinomas is in direct correlation with chronic inhalation of Cd, being in concordance with human data. Direct exposure of rats represents one of the main causes for the presence of prostatic proliferative lesions, including adenocarcinomas [5]. It was proven that Cd treatment in rats is responsible for the occurrence of tests and ventral prostate benign tumors [8].

Carcinogenic initiation consists in inheritable alteration in phenotype appear not only as a consequence of mutagenesis. Cd exposure affects cell proliferation, differentiation, apoptosis, cell signaling and other varieties of cellular activities, which can induce aberrant gene expression, errors in DNA methylation, blockage of apoptosis and disruption of differentiation. Cadmium presence into the human body can cause the aberrant activation of the expression for a large number of genes [6,9].

In actual context, public preoccupation against Cd contamination of waters and soil has great importance, due to various negative effects of such pollution. In contradiction to a bigger number of organic pollutants, heavy metals are generally refractory and it is not easy to degrade them or to use biological decontamination. In such context, heavy metals contaminate waters (surface or ground one) and surrounding soils [10,11,12]. Due to the high solubility and to the non-biodegradable nature of Cd ions, they can easily enter into the animal and human body through the food chain [12].

A natural reason for the temporary increase of environmental Cd concentration is represented by volcanic activity. Regularly, Cd is a constituent of ores together with Zn, Cu and Pd. Cd has a large usage in different industrial processes, being an anticorrosive agent, stabilizer in PVC products, pigment and a neutron absorber in the nuclear industry [3]. Main sources responsible for Cd contamination are anthropogenic, being represented by industrial wastewaters, mining operations, waste incineration and combustion of different coals and oils [13]. A problem is represented by the high Cd content of phosphate fertilizers, which lead to a constant increase of Cd concentration in soils used for intensive agriculture [3]. An additional issue related with Cd toxicity is represented by its accumulative character [13]. Due to his high toxicity, US Environmental Agency set the maximum content in drinking water at 0.005 mg·L^−1^, while World Health Organization has establish that the maximum content of Cd in drinking water should not exceed 0.003 mg·L^−1^ [3,13].

Considering how widespread the Cd contamination is and how limited the drinking water resources are, it will be mandatory to remove the heavy metals before discharging the wastewaters. Different techniques have been developed for metallic ion elimination from wastewaters, such as filtration, coagulation, chemical precipitation, ion exchange, solvent extraction, inverse osmosis, adsorption and bioremediation [11,12,13,14].

One of the most effective but prohibitive technologies is inverse osmosis; the main inconvenience being that the used membranes are so easily spoiled. Some other technologies such as chemical precipitation or solvents extraction can be applied with good results when larger quantities of metallic ions are present in the wastewater. The main disadvantage of these technologies is represented by the higher quantities of sludge generated during wastewater treatment. Adsorption and ion exchange are highly effective, promising widely applied methods [11,12]. From all these techniques, adsorption is the most popular due to its simple operation, higher efficiency and cost effectiveness. An ideal adsorbent material is a material with high surface area, good mechanical stability, higher adsorption capacity and is easily regenerated [7,11,13,15]. Solid phase extraction can play an important role into the removal of different pollutants from wastewater, by using different adsorbents (synthetic polymers, bio-adsorbents, silicates). Other techniques used for the removal of metallic ions from wastewater are involving ion exchange resins, magnetic nanoparticles [16,17,18], inorganic adsorbent materials such as: oxides, zeolites, fly ash, activated carbon [7,19,20,21], different nanoadsorbents and their composite materials [15,20,22,23,24,25,26] and some bionanocomposites adsorbents [27].

Activated carbon and ion exchange resins, which are extensively used in wastewater treatment, are relatively expensive, so by correlating this with the necessity of Cd removal, research was conducted in order to develop affordable adsorbent materials. This is why natural materials as well as wastes resulting from industry and agriculture should be used as adsorbents. Frequently, such adsorbents are designated as “cheap adsorbents” meaning that they have lower prices, being accessible because they are locally available. Kyzas and Kostanoglu describe such adsorbents as “green adsorbents” [25]. A possible limitation can be represented by lower adsorbtion capacity of such materials. By modifying organic and inorganic (synthetic and natural) adsorbent by functionalization with active groups containing nitrogen atoms, such limitations may be removed [28,29,30,31].

In last decades, adsorbent materials were functionalized using different compounds containing nitrogen groups, such as: tri-n-octylamine (TOA) [32], trioctylmethylammonium chloride [33], quaternary amine available commercially as Aliquat 336 [34], tetrabutylammonium dihydrogen phosphate [35], tetra butyl ammonium bromide [28] and tetra butyl ammonium chloride [7,36]. As a result that Cd presents a higher environmental toxicity, it is important to be able to remove it from wastewater before discharge [11].

All things considered, so far, our target was to produce a new adsorbent material with higher adsorption capacity and with higher selectivity for Cd removal from wastewater. This study introduces a new adsorbent material by using chitosan—a natural biopolymer—as support, which was functionalized with hexa-decyl-trimethyl-ammonium chloride (HDTACl).

## 2. Experimental Part

### 2.1. Obtaining the Biopolymer by Functionalization. Effect of Extractant Dosage

Chitosan (75%–85%, deacetylated) and oxalic acid dehydrate were purchased form Sigma Aldrich Chemicals (St. Louis, MO, USA). Chitosan was used as support and was functionalized with hexa-decyl-trimethyl-ammonium chloride using solvent impregnated resin dry method. Usage of hexa-decyl-tri-methyl-ammonium chloride as extractant is due to the presence of N containing groups.

In order to establish the extractant optimum dose for maximum adsorption capacities, we prepared adsorbent materials with different extractant quantities (0.05, 0.1, 0.15, 0. and 0.25 g of extractant for 1 g of chitosan). Chitosan and extractant solutions were kept in contact for 24 h, after that, they were washed and dried at 323 K for 24 h. Later, the obtained materials were used as adsorbents for Cd removal in order to establish the maximum quantity of extractant needed to maximize the adsorption capacity. Based on the obtained experimental data, we can conclude that the maximum quantity of extractant needed for functionalization of 1 g of chitosan is 0.1 g, because any further increase has no major influence on adsorption capacity. The efficiency of the functionalization process was 92%.

### 2.2. Characterization of the Functionalized Biopolymer

After preparation, the adsorbent materials were characterized by X-ray dispersion (EDX), scanning electron microscopy (SEM) and by Fourier transform infrared spectroscopy (FTIR), in order to confirm material functionalization, and in order to observe the morphology of the material surface.

#### 2.2.1. Batch Studies

All experimental studies were carried out using a Julabo thermostatic bath (Julabo Labortechnik, Seelbach, Germany), with shaker at a rotation speed of 200 rpm. The aim of present experiment was to establish the influence of pH, optimum ratio between adsorbent material and solution volume, temperature and initial concentration of Cd solution. All Cd solutions were prepared from a stock solution containing 1000 mg Cd(II)·L^−1^, which was obtained by dissolution Cd nitrate into DI water.

In order to determine how the solution pH is influencing the maximum adsorption capacity, 0.1 g of adsorbent material was scaled and brought in contact with 25 mL Cd solutions, having an initial concentration of 100 mg·L^−1^, and pH between 1 and 8. All samples were kept in contact for 2 h, at 200 rpm and 298 K.

The optimum ratio between adsorbent material quantity and volume of solution containing Cd ions was established by varying the quantity of adsorbent material (0.05, 0.1, 0.2, 0.3 and 0.4 g) used for same volume of solution (25 mL), with an initial concentration of 100 mg Cd(II)·L^−1^. All samples were kept in contact for 2 h at 298 K and 200 rpm.

Influences of contact time and temperature over the maximum adsorption capacity were established by using 0.1 g adsorbent material which was brought in contact with 25 mL solution having an initial concentration of 100 mg Cd(II)·L^−1^. Samples were kept in contact for different times (15, 30, 45, 60, 90 and 120 min) at three different temperatures (298, 308 and 318 K).

In order to establish the effect of initial concentration over maximum adsorption capacity of produced adsorbent material, we prepared solutions with different contents of Cd(II) ions (50, 100, 200, 300, 400, 500, 600, 700, 800 and 900 mg Cd(II)·L^−1^) by dilution of prepared stock solution. Further experiments were carried out at the optimum established pH, time and temperature (pH—4, time—60 min, temperature—298 K). Cd residual concentration into the filter solutions was determined by using atomic adsorption spectrophotometer (Varian AAS 280 S, Varian INC, Palo Alto, CA, USA).

Cd(II) quantity adsorbed on the mass unit of adsorbent material, expressed as mg·g^−1^, was calculated by using Equation (1):

(1) $$q_{e}=\frac{\left(C_{0}-\;C_{e}\right)\;V}{m}$$

where:

*q_e_*—the maximum absorption capacity (mg·g^−1^).
*C*_0_—initial concentration of cadmium (II) in solution (mg·L^−1^).
*C_e_*—the equilibrium concentration of cadmium (II) in solution (mg·L^−1^).
*V*—volume of the aqueous solution with cadmium (II) (L).
*m*—mass of the adsorbent (g).

#### 2.2.2. Sorption/Desorption Tests

A great importance for all adsorbent materials is represented by the sorption/desorption test, because is important to know if the used material can be regenerated and reused into a new adsorptive process.

Desorption tests were carried out by treating exhausted adsorbent material with 0.01 N HCl solution. Thereby, 1 g of exhausted material was kept in contact with 25 mL HCl solution for 4 h, after that the solution was filtered. The resulted solution was measured for the concentration of Cd(II) ions.

## 3. Results and Discussion

### 3.1. Characterization of the Functionalized Bio Sorbent

#### 3.1.1. X-ray Energy Dispersive Spectroscopy Analysis

**Figure 1:**
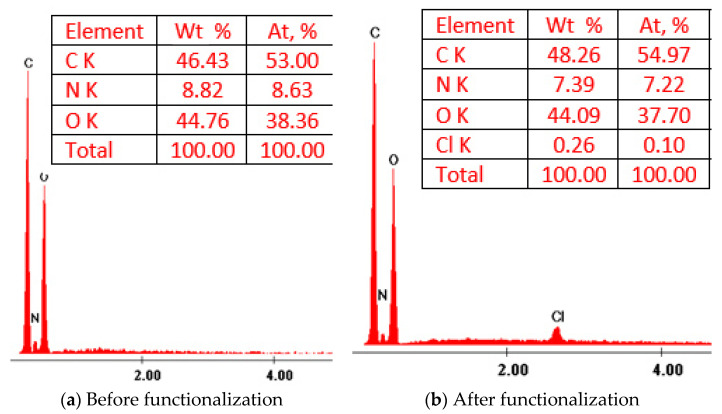
X-ray dispersion (EDX) spectra of unfunctionalized and functionalized bio material.

Functionalization of chitosan with hexa-decyl-trimethyl-ammonium chloride was confirmed by recording the EDX spectra (depicted in Figure 1). Analyzing the obtained spectra, we can observe the presence of peaks specific for chemical elements from the structure of bio-polymer (C, N, O), and the ones specific for the presence of the extractant onto the bio-polymer surface (N and Cl). Further, based on recorded EDX spectra, we determined the composition of raw and functionalized chitosan. The data are presented as the inset picture in Figure 1.

#### 3.1.2. Scanning Electron Microscopic Studies

**Figure 2:**
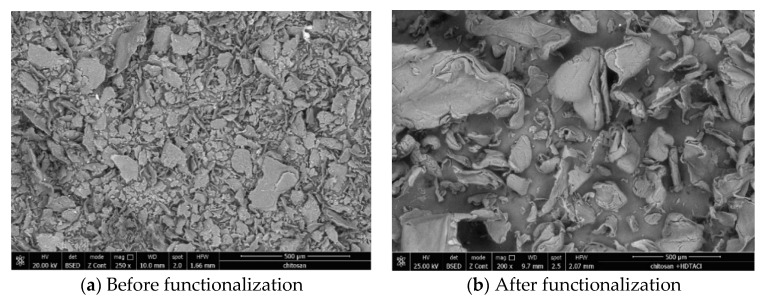
Scanning electron microscopy before (**a**) and after (**b**) biomaterial functionalized.

Further, raw chitosan and the functionalized one were characterized by recording the SEM pictures (images depicted in Figure 2).

From pictures depicted in Figure 2, we can observe that the chitosan surface suffers some morphological modification after the functionalization with hexa-decyl-trimethyl-ammonium chloride. Before functionalization, chitosan has a heterogeneous morphology with dispersed particles, presenting different sizes and geometry. After functionalization with HDTACl can be observed the presence of some molecular agglomeration related with the presence of some electrostatic forces between different molecules. Additionally, we observed the same heterogeneous morphology, with different particles size and more rounded geometrical shapes.

#### 3.1.3. Fourier Transform Infrared Spectroscopy Analysis

**Figure 3:**
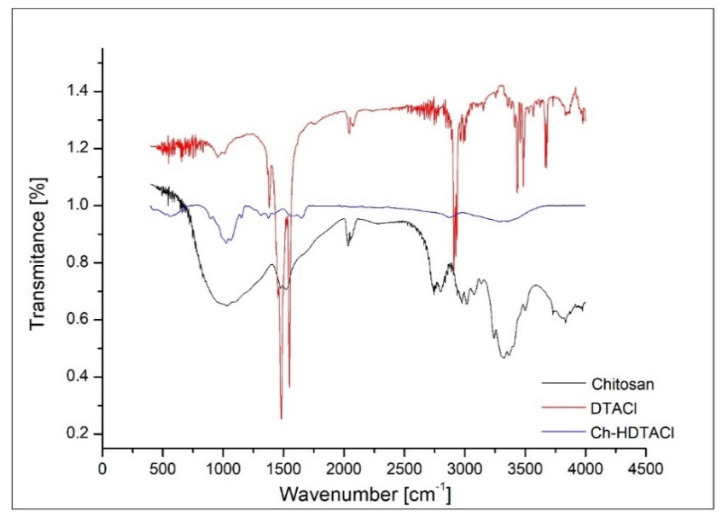
FTIR spectra.

Fourier transform infrared spectroscopy was used in order to confirm the functionalization of chitosan with used extractant. This confirmation is possible because, in the recorded spectra, we can identify adsorption bands specific for chitosan and for extractant. In Figure 3 is depicted the FTIR spectra obtained for raw chitosan and extractant and for functionalized chitosan.

Analyzing the FTIR spectra obtained for functionalized chitosan depicted in Figure 3, we observe the presence of adsorption bands located at 3300 and 3363 cm^−1^, bands associated with the stretching vibration of –OH and –NH groups [37]. Vibrations located at 2800 and 1500 cm^−1^ are specific for asymmetric stretching of –CH_3_ groups and/or for symmetric starching of –CH_2_ groups from extractant molecules. Adsorption band located at 2881 cm^−1^ is associated with the vibration of –CH groups, and bands located at 1651 and 1583 cm^−1^ are associated with the vibrations of groups –NH and –NH_2_. In addition, we observe the presence of two bands located at 1375 and 1317 cm^−1^, bands which are associated with the vibrations of –NH and –NH_2_ groups. Into the recorded FTIR spectra can observe the presence of two bands located at 1063 and 1030 cm^−1^, bands associated with the vibrations specific to the –C–O–C– bonds. Band located at 895 cm^−1^ is associated with the stretching vibration of –NH_2_ groups. Based on that, it can be said that in the FTIR spectra appear vibrations specific to a chitosan molecule and vibrations specific to the used extractant, so we can conclude that chitosan was functionalized with hex-adecyl-trimethyl-ammonium chloride.

### 3.2. Sorption Studies

All experimental data concerning the adsorption process on raw chitosan were included in the Supplementary Materials.

#### 3.2.1. Effect of pH over Cd Adsorption on Functionalized Chitosan

**Figure 4:**
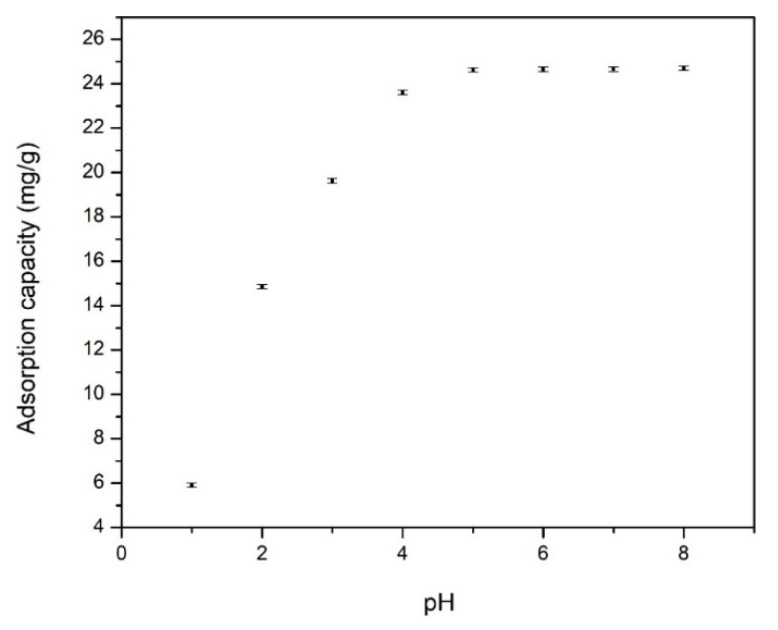
pH effect on adsorption capacity of Cd on functionalized chitosan.

In order to evaluate how the pH is influencing the adsorption of Cd ions, all experiments were carried out with the pH interval 1 to 8. Experimental studies were not conducted at pH higher then 8 because at pH over 8, Cd(II) precipitate [10,37]. Experimental adsorption capacities obtained when was evaluated the pH influence are depicted in Figure 4.

Analyzing the obtained experimental data presented in Figure 4, we can observe that the adsorption capacity increases with the increase of the pH value until pH 4. Further increase of the pH value has no significant influence on the maximum adsorption capacity. This behavior can be explained if we are considering that at lower pH the –NH_2_ groups are protonated, so the majority of adsorptive centers from the material surface are not free, explaining the lower adsorption capacity. When the pH is increasing, some groups –NH_2_ are not protonated because the proton concentration is decreasing. In this way, the competition between H^+^ and Cd(II) ions is not so harsh, leading to some increase of adsorption capacity of used adsorbent material. At pH over 8, the precipitation of Cd(II) ions is taking place which leads to some limitation of the adsorption capacity [37]. From the obtained experimental data, we can observe that the adsorption capacity remains constant at pH over 4; therefore, all further experimental studies were conducted at pH 4.

#### 3.2.2. Effect of Bio Adsorbent Dosage

Taking into account obtained experimental data, it can be stated that it is not justified to increase the adsorbent quantity in order to increase the ration S:L (ratio higher than 0.1 g adsorbent material: 25 mL Cd(II) solution) because the adsorption process efficiency does not change significantly. When the ratio S:L was 0.1 g with 25 mL of solution, the efficiency of the adsorption process was 92%.

#### 3.2.3. Effect of Contact Time, Temperature and Initial Cadmium Concentration

**Figure 5:**
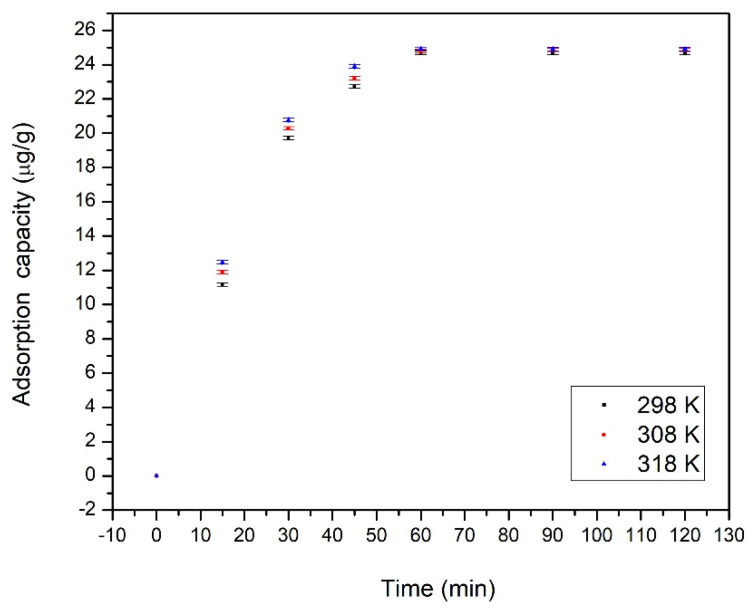
Effect of contact time and temperature on Cd adsorption on functionalized chitosan.

In Figure 5 is depicted the dependence between maximum adsorption capacity and contact time at three different temperatures.

From the data presented in Figure 5, we can observe that the maximum adsorption capacity increases with the increase of contact time and with the temperature increase. Based on the obtained data, we can observe that the adsorption capacity increases for the first 60 min, after that, further increase of contact time has no significant influence over the maximum adsorption capacity. Due to that, the optimum contact time used for further experiments was set to 60 min. It can also be observed that the increase of the temperature has almost no influence on the maximum adsorption capacity, so further experiments were carried out at room temperature (298 K).

**Figure 6:**
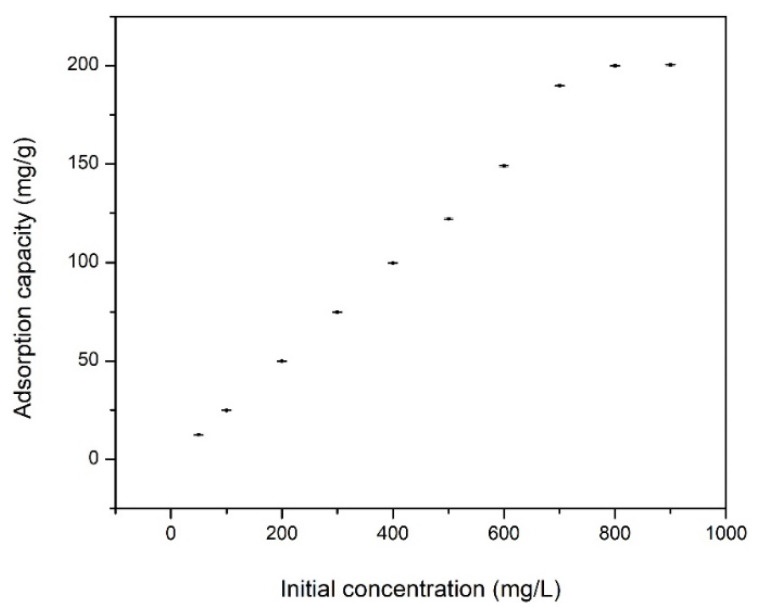
Effect of cadmium (II) initial concentration on Cd adsorption on functionalized chitosan.

In Figure 6 is presented the dependence between the adsorption capacity and the initial concentration of Cd ions (which vary from 25 to 900 mg·L^−1^).

Based on data depicted in Figure 6, we can observe that the adsorption capacity increases with the increase of the Cd(II) initial concentration, until it reaches a plateau. In this moment, the adsorbent material was saturated with Cd ions, this saturation occurs when the initial concentration of Cd ions has a value of 700 mg·L^−1^, which corresponds to a maximum adsorption capacity of 204.3 mg·g^−1^.

#### 3.2.4. Adsorptions Kinetics

In order to establish the mechanism associated with the adsorptive process, experimental data were fitted by using two different kinetic models: pseudo-first-order model and pseudo-second-order model.

Pseudo-first-order model was developed by Lagergren [38], and is described by relation:

(2) $$\ln(q_{e}-q_{t})=\ln q_{e}-k_{1}t$$

where

*q_e_*—the adsorption capacity at equilibrium (mg/g),
*q_t_*—is the adsorption capacity at time *t*,
*t*—the contact time (min) and *k*_1_ is the adsorption rate constant (1/min).

Pseudo-second-order kinetic model was developed by Ho and McKay [39] and is described by relation:

(3) $$\frac{t}{q_{t}}=\frac{1}{k_{2}q_{e}^{2}}+\frac{t}{q_{e}}$$

where

*q_e_*—the adsorption capacity at equilibrium (mg/g),
*q_t_*—the adsorption capacity at time *t*,
*t*—the contact time (min) and *k*_2_ the adsorption rate constant (g/mg∙min).

**Figure 7:**
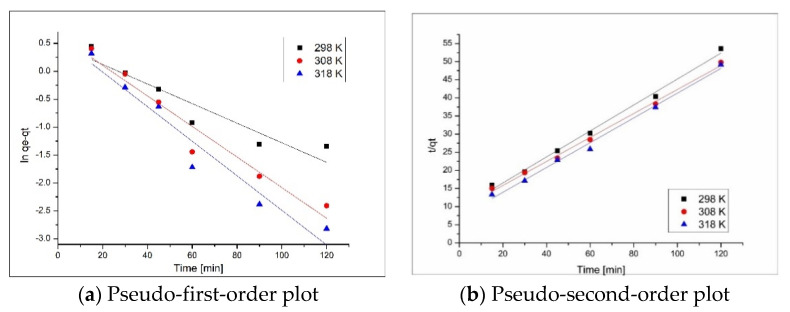
Kinetic models for Cd(II) adsorption onto Ch-HDTACl, at different temperatures.

In Figure 7 are presented the linearized forms of the used kinetics models—dependences between ln(*q_e_* − *q_t_*) and *t*/*q_t_* versus time. Form intercepts and slopes of the lines the kinetic parameters associated with the Cd(II) adsorption on the produced adsorbent are determined.

In Table 1 are presented the kinetic parameters associated with Cd(II) adsorption, parameters calculated using Lagergren and Ho and McKay kinetic models.

From data presented in Table 1, we can observe that the experimental data are better described by the pseudo-second-order kinetic model. This is confirmed by the calculated adsorption capacity, which has a value of 28.8 mg·g^−1^, really close to the experimental—24.7 mg·g^−1^. In addition, these results are sustained by the correlation coefficient *R*^2^ which has a value of 0.99, close enough to 1.

#### 3.2.5. Adsorption Isotherm Modelling

It is well known that when the equilibrium is attained the quantity of substance adsorbed by the material is equal with the quantity desorbed, and the concentration of the solution remains constant.

In order to better describe the Cd(II) adsorption process, experimental data were modeled using three different adsorption isotherms: Langmuir, Freundlich and Sips.

Nonlinear expression of Langmuir isotherm [40] is described by relation:

(4) $$q_{e}=\frac{q_{L}\;K_{L}\;C_{e}}{1+K_{L}\;C_{e}}$$

where:

*q_e_*—the maximum adsorption capacity (mg·g^−1^),
*C_e_*—the equilibrium concentration of cadmium (II) in solution (mg·L^−1^),
*q_L_*—Langmuir maximum adsorption capacity (mg·g^−1^),
*K_L_*—Langmuir constant.

Nonlinear expression of Freundlich adsorption isotherm is described by relation [41]:

(5) $$q_{e}{=K}_{F}C_{e}^{{1/n}_{F}}$$

where:

*q_e_*—the equilibrium adsorption capacity (mg·g^−1^),
*C_e_*—the equilibrium concentration of adsorbent in the solution (mg·L^−1^),
*K_F_* and *n_F_*—specific constants that are connected to the relative adsorption capacity of the adsorbent material and the intensity of adsorption.

Sips adsorption isotherm represents a combination between Langmuir and Freundlich adsorption isotherms, which is expressed by relation [42]:

(6) $$q_{e}=\frac{q_{s}\;K_{S}\;C_{e}^{1/n_{S}}}{{1+K}_{S}\;C_{e}^{1/n_{S}}}$$

where:

*q_S_—*the maximum adsorption capacity (mg·g^−1^),
*K_S_*—constant related to the adsorption capacity of the adsorbent,
*n_S_—*the heterogeneity factor.

**Figure 8:**
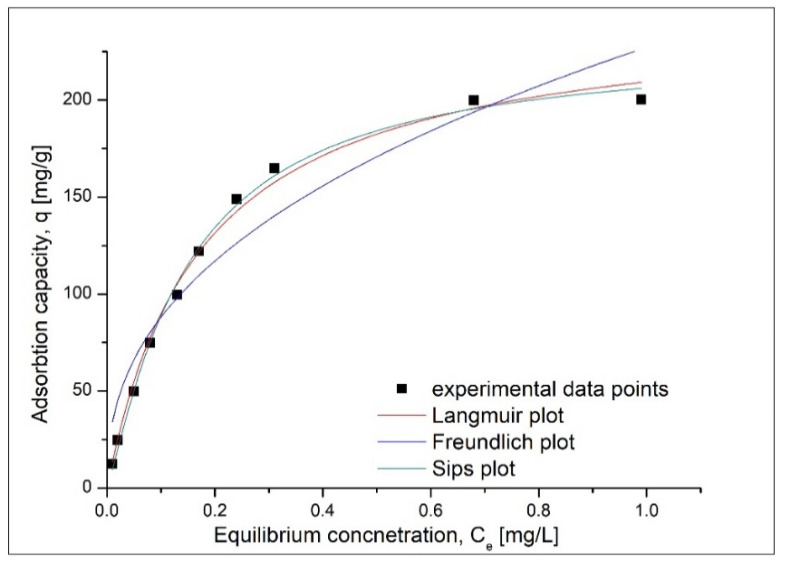
Adsorption isotherm obtained for Cd adsorption on Ch-HDTACl.

In Figure 8 are presented the adsorption isotherms which are used to describe the adsorption of Cd(II) ions onto the produced adsorbent material.

Based on the isotherm represented in Figure 8, we determined the parameters associated with adsorption isotherms (Table 2).

*q_m,exp_* is the maximum adsorption capacity experimentally obtained. Based on data presented in Table 2, we can conclude that the mechanism associated with the adsorption of Cd(II) ions onto Ch-HDTACI is better described by Sips model. This affirmation is sustained by the value close to 1 obtained for the correlation coefficient, in comparison with lower values obtained for other two used models. Other confirmation that the studied adsorption process is better described by Sips isotherm is represented by the value obtained for adsorption capacity, which have a value of 228.1 mg·g^−1^, relatively close to the experimental one—204.3 mg·g^−1^. As a result that the value of the coefficient ns is higher, the 1 can affirm that the studied adsorption process is a heterogeneous one.

In Table 3 are presented comparatively the maximum adsorption capacities obtained when different adsorbent materials were used for Cd(II) adsorption. By analyzing the data presented in Table 3, we can observe that by functionalizing chitosan with HDTACl, the maximum adsorption capacity is increasing from 6.63 mg·g^−1^ in the case of bare chitosan with 80% deacetylation at 204.3 mg·g^−1^. In addition, a good increase of maximum adsorption capacity in comparison with chitosan perlite was observed. Comparing the maximum adsorption capacity of modified chitosan, we can observe that the newly produced adsorbent material presents the maximum adsorption efficiency. Newly produced adsorbent material presents several advantages such as: relatively low price correlated with high stability, possibility to be reused for at least 20 times and the highest adsorption efficiency compared with similar chitosan adsorbents.

#### 3.2.6. Thermodynamic Analysis

Thermodynamic studies were carried out in temperature interval 298–318 K. Such studies were performed in order to get information about energetic exchanges associated with adsorption process in order to determine if the process is a spontaneous one. During the present study, we determined enthalpy, entropy and free Gibbs energy values. In order to establish if Cd adsorption on the new produced adsorbent is a spontaneous process, the value of Gibbs free energy was determined using the Gibbs–Helmholtz equation [47]:

ΔG°=ΔH°−T×ΔS°

where:

Δ*G*°—Gibbs free energy standard variation (kJ·mol^−1^),
Δ*H*°—enthalpy variation (kJ·mol^−1^),
Δ*S*°—entropy variation (J·mol^−1^∙K^−1^),
*T*—absolute temperature (K).

**Figure 9:**
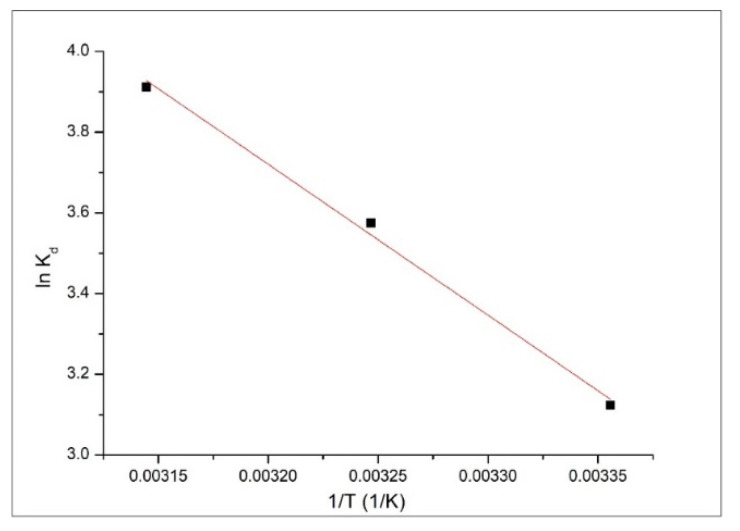
ln *K_d_* versus 1/T plot obtained for Cd adsorption on Ch-HDTACl.

Standard enthalpy and standard entropy variations can be calculated from the linear representation of van’t Hoff equation (ln *k_d_* versus 1/T—depicted in Figure 9):

$$ln\;K_{d}=\frac{\Delta S{}^{\circ}}{R}-\frac{\Delta H{}^{\circ}}{RT}$$

where:

*K_d_*—equilibrium constant,
Δ*H*°—enthalpy variation (kJ·mol^−1^),
Δ*S*°—entropy variation (J·mol^−1^∙K^−1^),
*T*—absolute temperature (K),
*R*—ideal gas constant (8.314 J·mol^−1^∙K^−1^).

Equilibrium constant is the ratio between the adsorption capacities (*q_e_*) obtained when the equilibrium was attained and the equilibrium concentration (*C_e_*):

$$K_{d}=\frac{q_{e}}{C_{e}}$$

Based on data depicted in Figure 9 were determined the values of thermodynamic parameters associated with Cd adsorption onto the studied adsorbent (values presented in Table 4).

Negative value of Gibbs free energy obtained for all working temperatures, correlated with the value obtained for enthalpy indicates that the Cd adsorption process on Ch-HDTACI is a spontaneous and exothermic process. Additionally from the data presented in Table 4, we can observe that the value of Gibbs free energy decreases with the increase of temperature indicating a beneficial effect of temperature increase. As a result that the differences between the values of Gibbs free energy obtained at different temperatures are not significant, from economical considerations it is recommended to work at 298 K. Due to the positive value of Δ*S*°, we can say that the distribution at the interface liquid/solid during adsorption is not a homogenous one [48].

Further, based on calculated enthalpy value, the value of activation energy associated with the studied adsorption process was determined. Activation energy was determined from the linear dependence of ln *k*_2_ versus 1/*t*.

Based on obtained experimental data, the activation energy for studied adsorption process has a value of 7.73 kJ·mol^−1^. As a result that the activation energy is lower than 8 kJ·mol^−1^, the adsorption process of Cd on Ch-HDTACl is a physical one.

**Figure 10:**
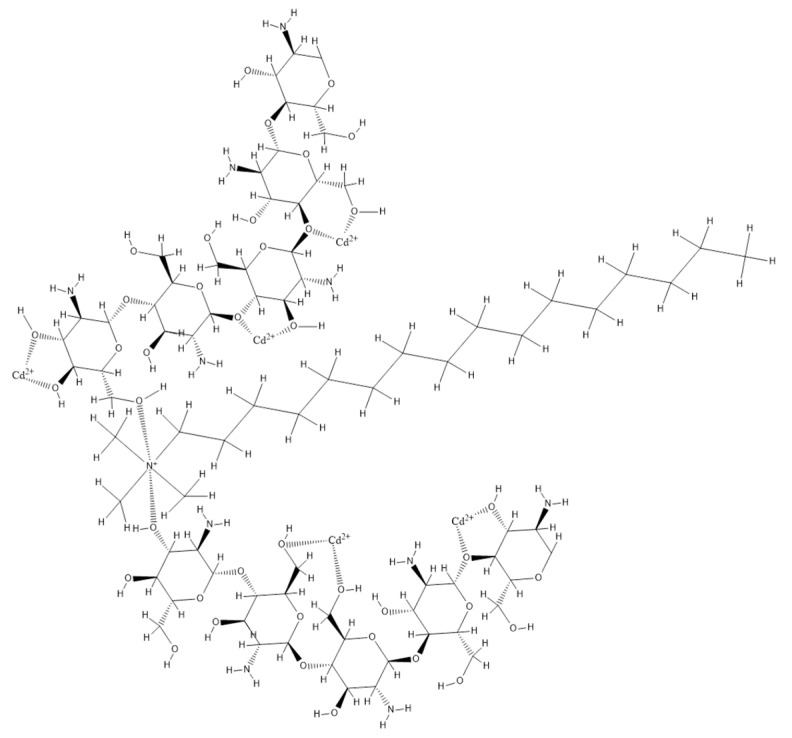
Proposed mechanism for chitosan functionalization and Cd adsorption on functionalized chitosan.

Based on all obtained data a possible mechanism for Cd adsorption on new prepared adsorbent material was proposed (Figure 10).

By functionalizing chitosan with HDTACl, the extractant molecule can be inserted between chitosan chains, which expose a higher number of –OH groups. Further, the Cd ions will form these groups by coordinative bonds with oxygen atoms.

#### 3.2.7. Sorption/Desorption Studies

Sorption/desorption studies reveal that the new produced adsorbent material can be reused for 20 adsorption cycles. During desorption tests, it was observed that after the first run the desorption degree was around 91%.

## 4. Conclusions

The present study reveals the possibility to produce a new adsorbent material by functionalizing a biopolymer (chitosan) with hexa-decyl-trimethyl-ammonium chloride by using SIR (Solvent Impregnated Resin) method, adsorbent material was produced by using a ration biopolymer: extractant = 10:1. SEM images reveal that the obtained adsorbent material presents a heterogeneous surface with small superficial pores. EDX spectra demonstrate that by using SIR, the functionalization of the used biopolymer with the hexa-decyl-trimethyl-ammonium chloride extractant was realized. In addition, functionalization of the chitosan was demonstrated by recording the FTIR spectra, which contain specific adsorption bands for support and for the used extractant.

Prepared adsorbent material was used for Cd(II) adsorption from aqueous solutions. Adsorption mechanism was revealed from kinetic, thermodynamic and equilibrium studies. From these studies, it was observed that Cd(II) adsorption is better described by pseudo-second-order kinetic model, being a physical and spontaneous adsorption. From equilibrium studies, it was observed that the adsorption mechanism is described by Sips adsorption isotherm. Maximum adsorption capacity evaluated based on Sips isotherm is 228.1 mg·g^−1^, being relatively close to the experimentally determined one—204.3 mg·g^−1^. It was also observed that the new produced adsorbent material can be used for several sorption/desorption cycles.

## Figures and Tables

**Table 1:** Kinetic parameters for the adsorption of cadmium (II) onto Ch-HDTACl at different temperatures.

**Pseudo-First Order**				
**Temperature (K)**	***q_e_*_,*exp*_** **(mg·g^−1^)**	***k*_1_** **(min^−1^)**	***q_e_*_,*calc*_** **(mg·g^−1^)**	***R*^2^**
298	24.70	0.0378	12.36	0.9783
308	24.87	0.0465	13.80	0.9829
318	24.92	0.0509	13.32	0.9834
**Pseudo-Second Order**				
**Temperature (K)**	***q_e,exp_*** **(mg·g^−1^)**	***k*_2_** **(g·mg^−1^∙min^−1^)**	***q_e,calc_*** **(mg·g^−1^)**	***R*^2^**
298	24.70	1510.83	28.81	0.9904
308	24.87	1670.06	28.57	0.9904
318	24.92	1838.49	28.18	0.991

**Table 2:** Parameters of the Langmuir, Freundlich and Sips adsorption isotherms.

**Langmuir Isotherm**			
*q_m,exp_* (mg·g^−1^)	*K_L_* (L·mg^−1^)	*q_L_* (mg·g^−1^)	*R* ^2^
204.3	5.76	245.8	0.9899
**Freundlich Isotherm**			
*K_F_* (mg·g^−1^)	1/*n_F_*		*R* ^2^
227.3	0.412		0.91612
**Sips Isotherm**			
*K_S_*	*q_S_* (mg·g^−1^)	1/*n_S_*	*R* ^2^
0.171	228.1	9.44	0.99551

**Table 3:** Adsorption capacities of some adsorbents cited in the literature.

Adsorbent	Adsorption Capacity, mg·g^−1^	Adsorption Conditions	Reference
Chitosan (80% deacetylation)	6.63	pH = 6.0, *T* = 313 K	[10]
Chitosan perlite	179.6	pH = 6.0, *T* = 313 K	[43]
Chitosan/activated carbon composite	52.6	pH = 6.0, *T* = 313 K	[44]
Chitosan coated cotton fiber	15.74	pH = 6.5, *T* = 313 K	[45]
Chitosan-phenylthiourea resin	120	pH = 5.0, *T* = 313 K	[46]
Ch-HDTACl	204.3	pH > 4.0, T = 298 K	Present paper

**Table 4:** Thermodynamic parameters for adsorption of cadmium (II) on the functionalized material.

Δ*H*° (kJ·mol^−1^)	Δ*S*° (J·mol^−1^∙K^−1^)	Δ*G*° (kJ·mol^−1^)			*R* ^2^
		298 K	308 K	318 K	
31.1	130.4	−6.4	−6.9	−7.3	0.9957

## Supplementary Materials

The following are available online at https://www.mdpi.com/1996-1944/13/23/5552/s1. Figure S1: pH influence over the maximum adsorption capacity of pure chitosan, Figure S2: Influence of initial concentration over maximum adsorption capacity, Figure S3: Influence of contact time and temperature over adsorption capacity of chitosan, Figure S4: Kinetic models for Cd (II) adsorption onto Chitosan at different temperatures, Figure S5: Adsorption isotherms obtained for Cd adsorption on chitosan, Figure S6: Log Kd-1/T plot obtained for Cd adsorption on Chitosan, Table S1: Kinetic parameters for the adsorption of Cd (II) onto chitosan, Table S2: Parameters of isotherm model for adsorption of Cd (II) onto chitosan, Table S3: Thermodynamic parameters for adsorption of Cd (II) onto chitosan.
