# Supplementary material for: Kinetics and Thermodynamics Studies for Cadmium (II) Adsorption onto Functionalized Chitosan with Hexa-Decyl-Trimethyl-Ammonium Chloride
Source: Materials (Basel). 2020 Dec 5;13(23):5552. doi: 10.3390/ma13235552 (PMC7730077; doi:10.3390/ma13235552)
Supplement: Supplementary file 1 [file materials-13-05552-s001.pdf]

materials

MDPI

Supplementary Materials: Kinetics and Thermodynamics Studies for Cadmium (II) Adsorption onto Functionalized Chitosan with Hexa-Decyl-Trimethyl-Ammonium Chloride

Cristina Ardean, Mihaela Ciopec, Corneliu Mircea Davidescu *, Petru Negrea * and Raluca Voda

Faculty of Industrial Chemistry and Environmental Engineering, Politehnica University of Timisoara, 2 Piata Victoriei, RO 300006 Timisoara, Romania; cristina.ardean@student.upt.ro (C.A.); mihaela.ciopec@upt.ro (M.C.); raluca.voda@upt.ro (R.V.)

* Correspondence: corneliu.davidescu@upt.ro (C.M.D.); petru.negrea@upt.ro (P.N.)

Raw chitosan was used as adsorbent for Cd removal from aqueous solutions. Similar with procedure used for characterization of new produced material, pure chitosan was used as adsorbent material into the batch studies, which were carried out using a Julabo thermostatic bath, with a shaker at a rotation speed of 200 rpm. Aim of present experiment was to establish the influence of pH, optimum ratio between adsorbent material and solution volume, temperature, and initial concentration of Cd solution. All Cd solutions were prepared from a stock solution containing 1000 mg Cd(II)·L^{-1}, which was obtained by dissolution Cd nitrate into DI water.

pH influence was evaluated by measuring 0.1 g of pure chitosan, which has been brought in contact with 25 mL Cd solutions with an initial concentration of 100 mg·L^{-1}, with pH between 1 and 8. All samples were kept in contact for 2 h at 200 rpm and 298 K. Obtained experimental data are presented in Figure S1.

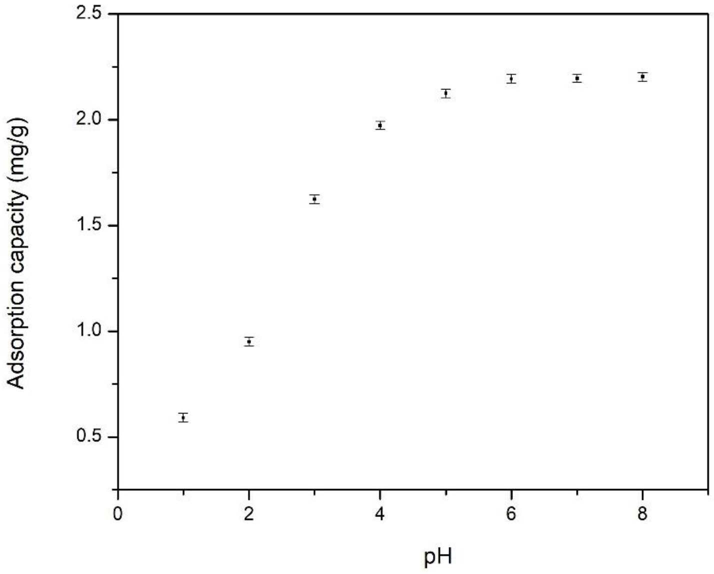

Figure S1. pH influence over the maximum adsorption capacity of pure chitosan.

From data presented in Figure S1 can observe that the maximum adsorption capacity present a sharp increase with pH increase until pH = 4 (from 0.5 mg·g^{-1} at pH = 1 to 2.0 mg·g^{-1}), further increase of solution pH leads at no significant increase of maximum adsorption capacity of chitosan.

Further has been evaluated the influence if Cd initial concentration over the maximum adsorption capacity, recorded data being presented in Figure S2.

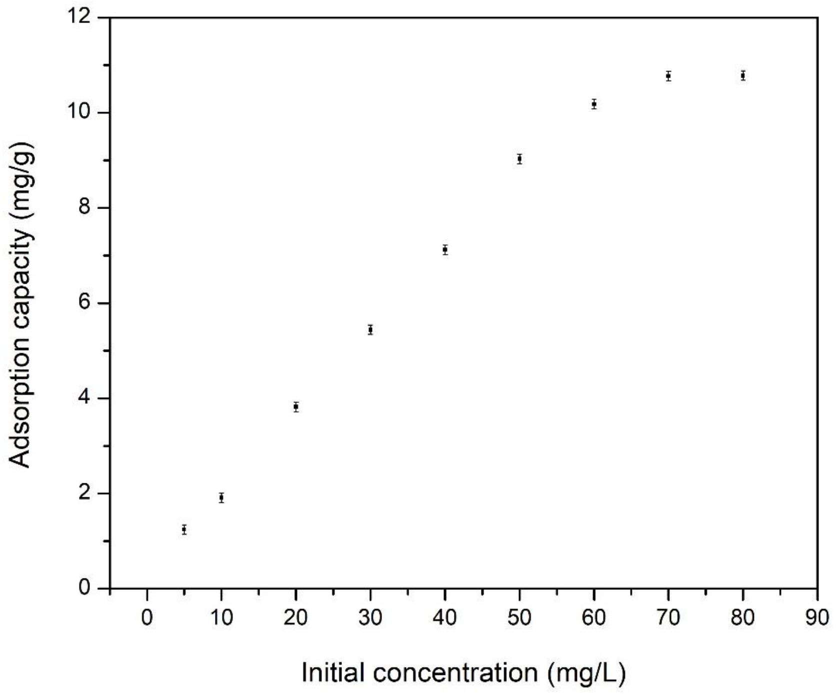

Figure S2. Influence of initial concentration over maximum adsorption capacity.

From data presented in Figure S2 can observe that the maximum adsorption capacity is increasing with the increase of the initial concentration. When the initial concentration of Cd ions is reaching 80 mg·L^{-1}, the adsorption capacity of chitosan is reaching his maximum value of 10 mg·g^{-1}, any further increase of Cd initial concentration lead at no further increase of maximum adsorption capacity of chitosan.

Influences of contact time and temperature over the maximum adsorption capacity were established by using 0.1 g adsorbent material which was brought in contact with 25 mL solution having an initial concentration of 100 mg·Cd(II)·L^{-1}. Samples were kept in contact for different time (15, 30, 45, 60, 90 and 120 min) at three different temperatures (298, 308, and 318 K).

In order to establish the effect of initial concentration over maximum adsorption capacity of produced adsorbent material were prepared solutions with different content of Cd(II) ions between 15 and 90 mg Cd(II)·L^{-1} by dilution of prepared stock solution. Obtained data are depicted in Figure S3.

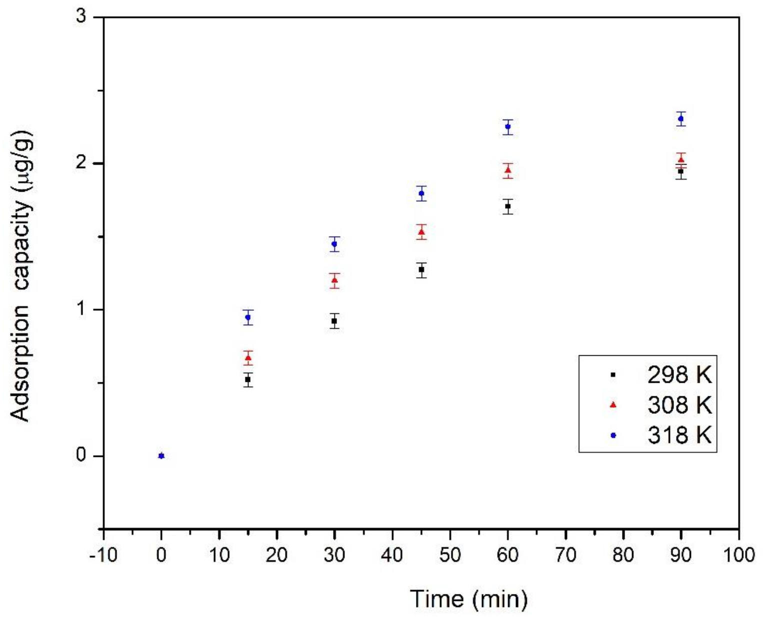

Figure S3. Influence of contact time and temperature over adsorption capacity of chitosan.

From data presented in Figure S3 can observe that the adsorption capacity of chitosan is increasing with increase of the contact time, until a maximum adsorption capacity was obtained at 60 minutes. Any further increase of contact time leads at no increase of the adsorption capacity. Regarding the influence of temperature, from data presented in Figure S3 can observe the beneficial effect of temperature increase, as was expected. Further experiments were carried out at optimum established pH, time and temperature (pH—4, time—60 min, temperature—298 K).

Adsorptions Kinetics

In order to establish the mechanism associated with the adsorptive process experimental data were fitted by using two different kinetic models (Figure S4): pseudo-first-order model and pseudo-second-order model (presented in detail in main paper).

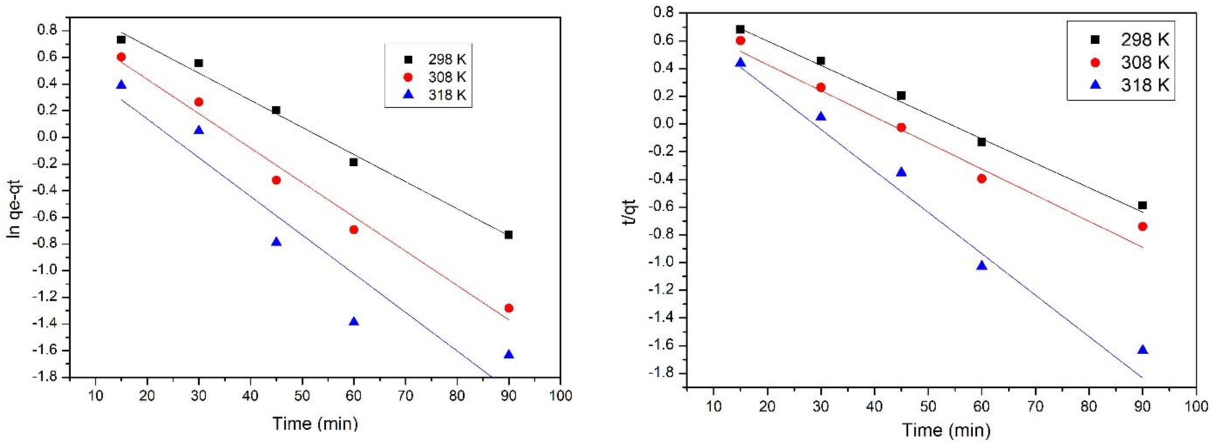

(**a**) Pseudo-first-order model (**b**) Pseudo-second-order model

Figure S4. Kinetic models for Cd (II) adsorption onto Chitosan at different temperatures.

Form intercepts and slopes of the lines the kinetic parameters associated with the Cd(II) adsorption on the produced adsorbent are determined (obtained parameters being presented in Table S1).

Table S1. Kinetic parameters for the adsorption of Cd (II) onto chitosan.

**Pseudo-First Order**				
**Temperature (K)**	$q_{e,exp}$ **(mg·g⁻¹)**	k_1 **(min⁻¹)**	$q_{e,calc}$ **(mg·g⁻¹)**	R^2
298	1.71	0.0137	1.46	0.9802
308	1.95	0.0174	1.51	0.9205
318	2.25	0.0320	1.79	0.9167
**Pseudo-Second Order**				
**Temperature (K)**	$q_{e,exp}$ **(mg·g⁻¹)**	k_2 **(g mg⁻¹·min⁻¹)**	$q_{e,calc}$ **(mg·g⁻¹)**	R^2
298	1.71	0.0902	1.72	0.9951
308	1.95	0.2539	2.04	0.9946
318	2.25	0.7016	2.56	0.9967

From data presented in Table S1 can conclude that the Cd adsorption onto the chitosan is better described by the pseudo-second-order model.

Adsorption Isotherm Modelling

It is well known that when the equilibrium is attained the quantity of substance adsorbed by material is equal with the quantity desorbed, and the concentration of the solution remains constant. In order to better describe Cd(II) adsorption process, experimental data were modelled using three different adsorption isotherms: Langmuir, Freundlich and Sips (data presented in Figure S5).

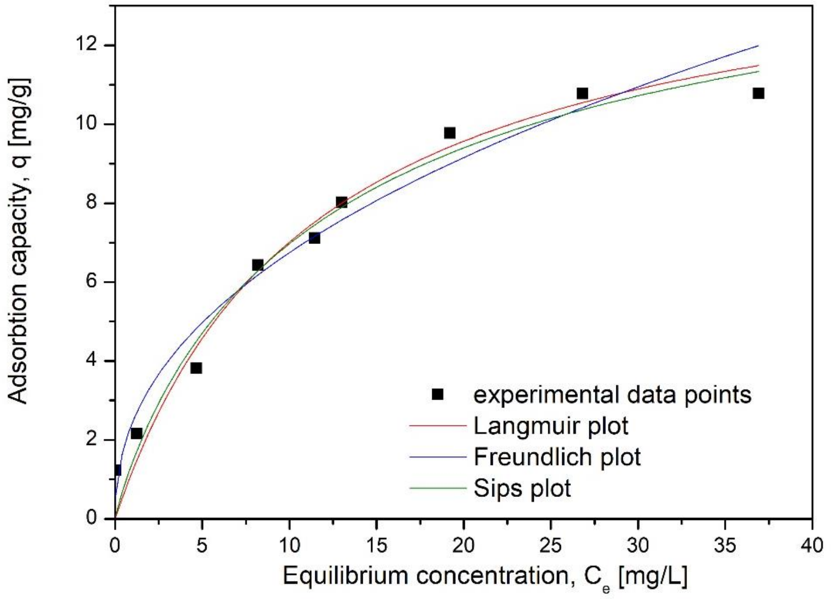

Figure S5. Adsorption isotherms obtained for Cd adsorption on chitosan.

Based on data depicted in Figure S5 were determined the specific parameters for each isotherm used to model the experimental data (parameters depicted in Table S2).

Table S2. Parameters of isotherm model for adsorption of Cd (II) onto chitosan.

**Langmuir Isotherm**			
$q_{m,exp}$ **(mg/g)**	K_L **(L/mg)**	q_L **(mg/g)**	R^2
10.77	0.087	15.05	0.9467
**Freundlich isotherm**			
K_F **(mg/g)**	$1/n_F$		R^2
2.44	0.4406		0.9240
**Sips isotherm**			
K_S	q_S **(mg/g)**	$1/n_S$	R^2
0.100	11.71	0.1	0.9731

From parameters presented in Table S2 can observe that the Cd adsorption onto the chitosan is better described by Sips isotherm, similar with the adsorption of Cd on new prepared adsorbent.

Thermodynamic Analysis

Thermodynamic studies were carried out in temperature interval 298–318 K. Ln K_d versus $1/T$ plot obtained for Cd adsorption on Chitosan were shown in Figure S6. Such studies were performed in order to get information about energetic exchanges associated with adsorption process, in order to determine if the process is a spontaneous one. During present study were determined enthalpy, entropy, and free Gibbs energy values, presented in Table S3.

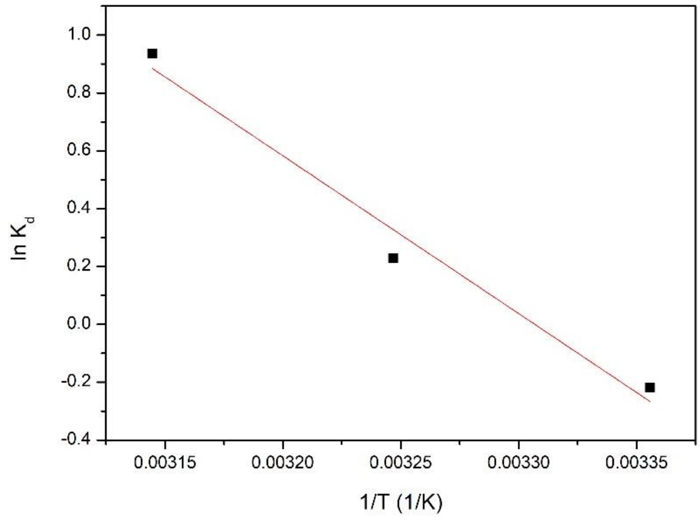

Figure S6. ln K_d versus $1/T$ plot obtained for Cd adsorption on Chitosan.

Table S3. Thermodynamic parameters for adsorption of Cd (II) onto chitosan.

$\Delta H°$ **(kJ/mol)**	$\Delta S°$ **(J/mol·K)**	$\Delta G°$ **(kJ/mol)**			R^2
		**298 K**	**308 K**	**318 K**	
3.28	18.5	−2.2	−2.4	−2.5	0.9998

Negative value of free Gibbs energy obtained for all working temperatures, correlated with the value obtained for enthalpy indicates that the Cd adsorption process on Chitosan is a spontaneous and exothermic process. Also from data presented in Table S3 can observe that the value of free Gibbs energy decreases with the increase of temperature indicating a beneficial effect of temperature increase. But, because the differences between the values of free Gibbs energy obtained at different temperatures are not significant, from economic considerations is recommended to work at 298 K. From the linear dependence of ln K_2 versus $1/t$ has been determined the activation energy for Cd

adsorption on chitosan. From obtained experimental data was obtained for the studied adsorption process a value of 18.3 kJ/mole.

Publisher's Note: MDPI stays neutral with regard to jurisdictional claims in published maps and institutional affiliations.

© 2020 by the authors. Licensee MDPI, Basel, Switzerland. This article is an open access article distributed under the terms and conditions of the Creative Commons Attribution (CC BY) license (http://creativecommons.org/licenses/by/4.0/).
